# Diabetes and Risk of Tuberculosis Relapse: Nationwide Nested Case-Control Study

**DOI:** 10.1371/journal.pone.0092623

**Published:** 2014-03-24

**Authors:** Pin-Hui Lee, Hui-Chen Lin, Angela Song-En Huang, Sung-Hsi Wei, Mei-Shu Lai, Hsien-Ho Lin

**Affiliations:** 1 Centers for Disease Control, Taipei, Taiwan; 2 Graduate Institute of Epidemiology and Preventive Medicine, College of Public Health, National Taiwan University, Taipei, Taiwan; 3 Community Medicine Research Center and Institute of Public Health, National Yang-Ming University, Taipei, Taiwan; Fundació Institut d'Investigació en Ciències de la Salut Germans Trias i Pujol. Universitat Autònoma de Barcelona. CIBERES, Spain

## Abstract

The aim of this study was to investigate the association between diabetes mellitus (DM) and tuberculosis (TB) relapse using the nationwide TB registry in Taiwan. We conducted a case-control study nested within a nationwide cohort of all incident cases of pulmonary TB that were notified during 2006–2007 and had completed anti-TB treatment. The relapse of TB was confirmed by bacteriological or pathological findings. For each relapse case, one control was selected from the study cohort matching by time since treatment completion. DM status was ascertained by medical chart review and cross-matching with the National Health Insurance claims database. A total of 305 cases of relapse were identified after a median follow-up of 3 years (relapse rate: 488 per 100,000 person-year; 95% confidence interval (CI): 434–546). Presence of DM during previous anti-TB treatment was 34.0% and 22.7% in cases and controls, respectively. After adjusting for other potential confounders, DM was associated with increased risk of TB relapse (adjusted odds ratio: 1.96, 95% CI: 1.22–3.15). Only one-third of the DM-TB patients in our study received glycaemic monitoring using HbA1c during anti-TB treatment. Presence of DM was independently associated with risk of TB relapse. TB programs should seriously consider rigorous glucose control in DM-TB patients.

## Introduction

Despite effective antimicrobial chemotherapy, relapse of tuberculosis (TB) after treatment completion remains a major challenge for TB control. Globally, an estimated 300,000 patients had relapse of TB in 2011 [1]. The incidence of recurrent TB in those who completed previous treatment can be 30 times higher than the incidence of TB in the general population [2]. Relapse of TB is associated with increased risk of drug resistance because of previous exposure to first-line anti-TB chemotherapy. In the recent global surveillance for drug-resistant TB, it was estimated that 7.9% of relapse cases were multidrug resistant (MDR, isolates resistant to both isoniazid and rifampicin) TB [3]. Relapse patients also have lower cure rate than incident TB and encounter more side effects during treatment with expensive second-line drugs [4].

Diabetes mellitus (DM) has become increasingly prevalent in low and middle-income countries where TB is most concentrated [5]. Despite the compelling evidence that DM increases the risk of incident TB, studies on the association between DM and TB relapse were relatively few and limited by small sample size and confounding by other factors [6], [7]. Better understanding of the determinants of TB relapse helps to identify those at high risk of relapse disease and promises to reduce the disease burden through risk factor intervention. The direct policy implication of clarifying the association between DM and TB relapse would be screening DM patients for TB relapse and better clinical management of DM in patients with TB. We believe the importance of investigating the relation between DM and TB relapse is twofold. First, if the association is found to be strong, DM patients who complete the incident TB treatment should be carefully monitored for the possibility of TB relapse. Second, co-management of DM-TB patients could be an important strategy to reduce TB relapse. We conducted a nested case-control study to investigate the association between DM status during anti-TB treatment and subsequent relapse of TB using data from national TB registry in Taiwan.

## Materials and Methods

### Study population

We conducted a case-control study nested within the national cohort of all incident cases of pulmonary TB who were notified to Taiwan TB registry during 2006–2007 and had completed anti-TB treatment. TB has been a mandatory notified disease in Taiwan for decades. Physicians are regulated by law to report TB patients whether they are incident or recurrent cases. Taiwan Centers for Disease Control maintains a web-based electronic database for the national TB registry which includes the patient's unique individual identifier and relevant bacteriological and clinical information. In Taiwan, the health care service for TB is reimbursed through the National Health Insurance (NHI), which covers over 99% of the population. To improve the notification of TB, the Bureau of National Health Insurance introduced the policy of “no-notification, no-reimbursement” in 1997. A recent analysis using the cross-matched database of TB notification and NHI reimbursement found that over 96% of TB patients were notified to TB registry [8].

Following the national TB guideline, a case of pulmonary TB was defined by positive sputum smear or sputum culture or, in the absence of bacteriological evidence, positive chest X-ray compatible with pulmonary TB plus clinical improvement after anti-TB treatment [9]. Of the 27,878 incident cases of pulmonary TB during the study period, we excluded cases who defaulted (n = 72), transferred out (n = 31), died during anti-TB treatment (n = 5,531), and were still on treatment or found to be MDR-TB (n = 305). We adopted the WHO definition for TB treatment outcomes [10]. After exclusion, 21,939 patients were followed up for development of TB relapse until December 31st of 2010.

### Selection of cases and controls

**Figure 1:**
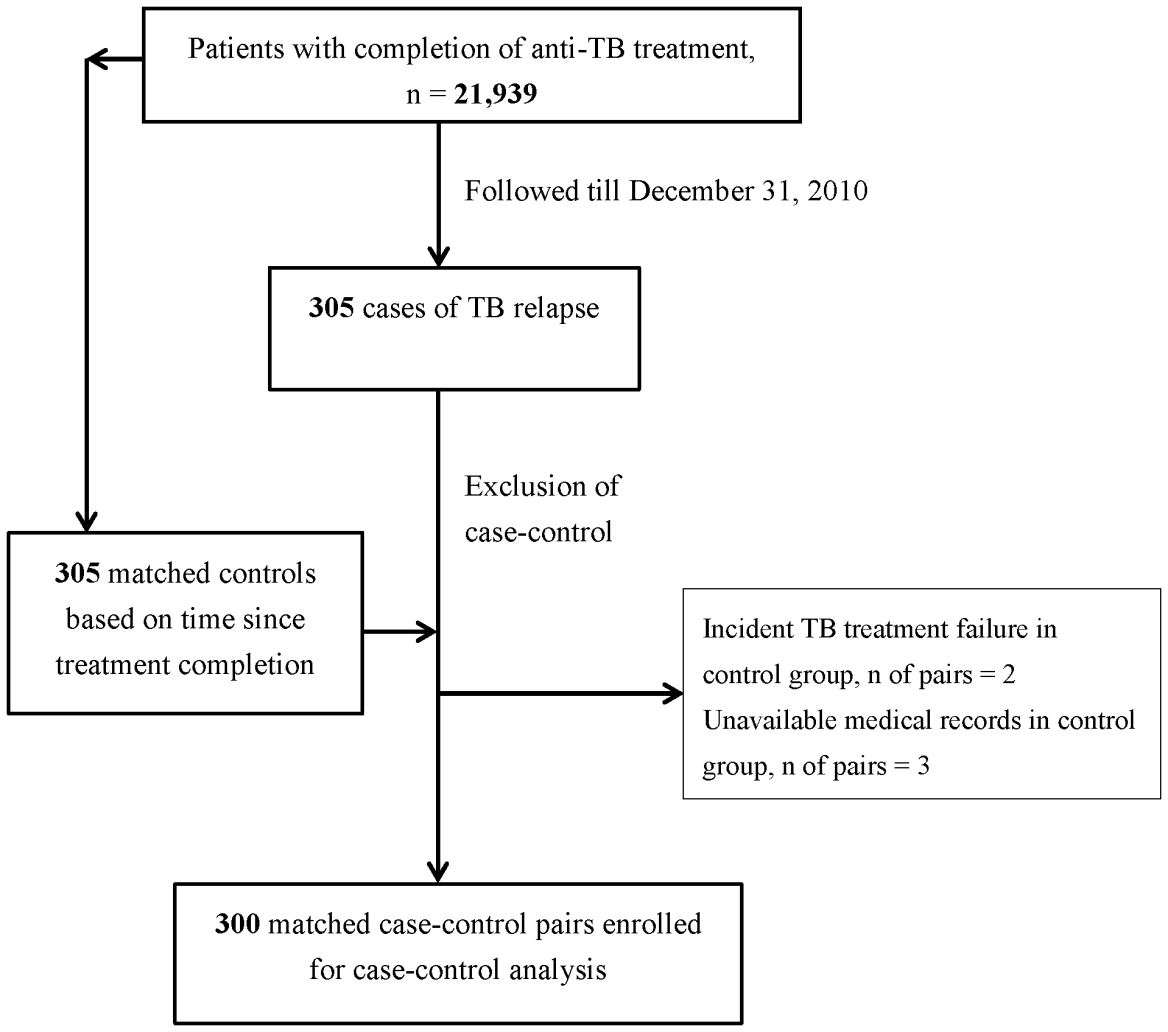
Flow diagram for the enrollment of study participants in the nested case-control study.

In the nested case-control study, we included all TB relapse cases from the study cohort during the follow-up period (n = 305). Relapse was defined as re-notification to the national TB registry with bacteriological (smear or culture positive) or pathological finding compatible with active TB after completion of previous anti-TB treatment. We selected controls from the study population with 1∶1 ratio to relapse cases using density (person-time) sampling and matching on time since completion of previous anti-TB treatment. We conducted medical chart review for the 305 matched pairs in the health care facilities where anti-TB treatment was given. Chart review was performed by four medical officers from Taiwan Centers for Disease Control. Five matched pairs were further excluded because the controls were found to have not completed previous anti-TB treatment or because the medical chart could not be obtained (Fig. 1). The final sample size was 300 matched pairs.

### Measurement of diabetes mellitus and other covariates

The DM status of study participants was ascertained by using the patient's unique individual identifier from the TB registry to cross-match with the National Health Insurance (NHI) claims database and medical chart review in the health care facilities where anti-TB treatment was given. NHI is a single-payer compulsory health insurance with coverage over 99% of the population in Taiwan [11]. The cross-matching of NHI and TB registry was performed by the staff in the Collaboration Center of Health Information Application, Ministry of Health and Welfare. All patients' record and information was anonymized and de-identified prior to the analysis. The definition of DM includes any of the following conditions before completing previous anti-TB treatment: (1) use of DM medication documented in the NHI claims database; (2) use of DM medication (either in the same health care facility or in other facilities) documented in the medical chart; (3) DM diagnosis (ICD-9-CM of 250) with fasting sugar ≥7.0 mmol/L in the medical chart. We considered the following drug categories as DM medication: sulfonylureas, biguanides, alpha-glucosidase inhibitors, thiazolidinediones, meglitinides, and insulin. Hemoglobin A1c (HbA1c) reflects the average blood glucose level over prior 2 to 3 months and is a strong predictor for diabetes complications [12]–[14]. We therefore measured the frequency of HbA1c exam among those with DM as an indicator for better glucose monitoring and access to diabetes care.

Information on other covariates was obtained from the national TB registry and medical chart review. The TB registry contained essential information of notified cases including unique individual identifier, the number of medical chart with records of anti-TB treatment, TB care facility, age at incident TB diagnosis, sex, indigenous population (yes/no), bacterial and pathological evidence of TB disease, notification date of recurrent episode, and the proportion of treatment received on directly observed therapy (DOT). We extracted the following information through medical chart review: history of smoking, history of alcohol use, weight at the time of TB diagnosis, HIV status, cancer, end-stage renal disease (ESRD) defined by an estimated glomerular filtration rate of <15 ml/min/1.73 m [15], coexisting extra-pulmonary TB, cavitation on chest X-ray within one month of initiating anti-TB treatment, results of drug susceptibility testing at the incident TB episode (susceptibility to isoniazid, rifampicin, ethambutol, and streptomycin), and use of suboptimal TB regimen (intensive phase with combination of isoniazid, rifampicin, ethambutol, and pyrazinamide of less than 2 months, continuation phase with combination of isoniazid, rifampicin and ethambutol of less than four months in patients with pan-susceptible strain, or treatment duration of less than 18 months in those with rifampicin resistance) [9].

### Power calculation

At the significance level of 5% and with 300 pairs of cases and controls, assuming a 20% prevalence of DM among incident TB patients, the statistical power was estimated to be 99.5% to detect an odds ratio (OR) of 2.0 and 75.4% to detect an OR of 1.5 [6], [16].

### Statistical analyses

We used conditional logistic regression to estimate the univariable and multivariable-adjusted OR and 95% confidence interval (CI) for the association between DM status and subsequent TB relapse. In the multivariable analysis, we adjusted for all potential risk factors for TB based on literature review (see ***Measurement of diabetes mellitus and other covariates***) [17]–[21]. For investigating the association between glycaemic control and TB relapse, we used the frequency of HbA1c testing during anti-TB treatment as a proxy and estimated the OR of TB relapse among patients with DM who tested at least once HbA1c, without any measurement of HbA1c, and those without DM. In order to explore the most relevant exposure to DM and relapse of TB, the DM definition was ascertained with different exposure windows: (1) presence of DM before completing previous anti-TB treatment (main analysis); (2) presence of DM before completing previous anti-TB treatment plus the first year after treatment completion; (3) presence of DM any time before the date of TB relapse. We tested whether the association between DM and TB recurrence might be modified by the following factors: (1) age (<40, 40–59, ≥60 years), (2) follow-up time to recurrence (<1 year, 1–1.9 years, and ≥2 years). We added cross-product terms to the multivariable model to estimate the adjusted OR of DM in different age groups and different follow-up periods after anti-TB treatment completion for relapse of TB. In order to test for effect modification, we compared models with and without the cross-product terms using the likelihood ratio test. Because of the small proportion of case-control pairs with missing covariates (16% of all pairs), we only included the pairs with complete information in the main multivariable analysis [22]. All statistical analyses were performed using SAS version 9.2 software packages (SAS Institute Inc., Cary, NC).

### Ethical approval

This study was approved by the ethics committee of Taiwan Centers for Disease Control (IRB no. : TwCDCIRB100023). Because the retrospective review of the TB registry and medical records, inform consents were not available. All patients' record and information was anonymized and de-identified prior to analysis.

## Results

Among the 21,939 cases of incident TB who completed anti-TB treatment, 305 cases (1.4%) developed relapse of TB during the median follow-up period of 3.0 years (interquartile range (IQR) 2.7–3.7 years). The median time from treatment completion to relapse was 1.4 years (IQR 0.7–2.2 years). None of the cases had a second episode of relapse. The incidence rate of TB relapse in this nationwide cohort was 488 cases per 100,000 person-years (95% CI: 434–546).

Three hundred matched case-control pairs were included in the analysis of DM and relapse of TB. The median age at previous TB diagnosis was 58.7 years (IQR: 41.8–75.3); the distribution of age did not differ between cases and controls (Table 1). Eighty six percent of relapse cases had bacteriological evidence for active TB disease. The prevalence of DM was higher among cases than controls (34.0% vs. 22.7%). Compared to controls, cases were more likely to be indigenous people, alcohol users, and smokers. Cases were also more likely to have cancer and ESRD, to present with initial cavitation, and to receive suboptimal TB regimen (Table 1).

**Table 1:** Characteristics of cases and controls among the study participants.

	Case (n = 300) N (%)	Control (n = 300) N (%)
DM	102 (34.0)	68 (22.7)
Age (year)		
<40	56 (18.7)	75 (25.0)
40–59	90 (30.0)	86 (28.7)
≥60 year	154 (51.3)	139 (46.3)
Body weight (kilogram) *		
<50	97 (32.3)	70 (23.3)
50–69	165 (55.0)	179 (59.7)
≥70	38 (12.7)	27 (9.0)
Sex, male	227 (75.7)	205 (68.3)
Indigenous population	31 (10.3)	6 (2.0)
History of alcohol use	78 (26.0)	42 (14.0)
History of smoking	146 (48.7)	116 (38.7)
Cancer	36 (12.0)	24 (8.0)
ESRD	6 (2.0)	4 (1.3)
Coexisting of extra-pulmonary lesion	51 (17.0)	48 (16.0)
Drug susceptibility†		
Pan-susceptible	147 (58.6)	133 (53.0)
Mono-drug resistant	18 (7.2)	11 (4.4)
Poly-drug resistant	9 (3.6)	4 (1.6)
Culture negative or susceptibility test not performed	77 (30.7)	103 (41.0)
Cavitation on initial CXR‡	65 (21.7)	59 (19.7)
Suboptimal regimen	69 (23.0)	48 (16.0)
DOT ≥60%	87 (29.0)	66 (22.0)

*Missing inf°rmation in 24 case-control pairs

† Susceptibility of isolates to isoniazid, rifampicin, ethambutol, and streptomycin at the incident TB episode

‡ Missing information in 25 case-control pairs

Values are numbers (percentages).

**Figure 2:**
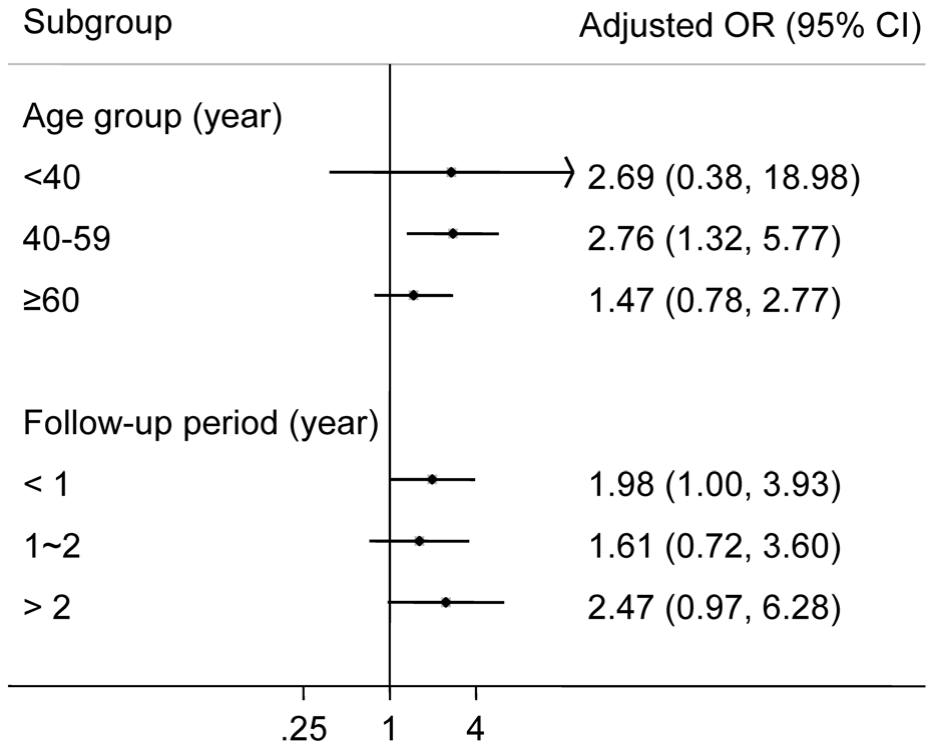
Adjusted odds ratio for the association between DM and TB relapse, by age-group and follow-up period.

DM during previous anti-TB treatment was associated with relapse of TB both in the univariable analysis (crude OR: 1.67, 95% CI: 1.18–2.38) and the multivariable analysis (adjusted OR: 1.96, 95% CI: 1.22–3.15) (Table 2). In the multivariable analysis we included only 251 case-control pairs because of missing information in body weight and results of chest X-ray. We conducted a separate analysis using all 300 case-control pairs and adjusting for all covariates except for body weight and results of chest X-ray; the association was slightly attenuated but remained statistically significant (adjusted OR: 1.55, 95% CI: 1.04–2.38). In the subset analysis of case-control pairs of only bacteriologically confirmed cases (213 case-control pairs), the association was attenuated with wider confidence interval (adjusted OR: 1.62, 95% CI: 0.96–2.73). When we used different exposure windows for DM definition, the increased risk of DM on TB relapse was consistently observed. The OR was largest when the exposure window was defined by the period before completing previous anti-TB treatment plus the first year after treatment completion (adjusted OR: 2.40, 95% CI: 1.49–3.86, Table 3). In the age-specific analysis, the adjusted OR for DM and TB relapse seemed to be lower in those older than 60 compared to that in other age groups (Fig. 2), though the test of effect modification by age was not statistically significant (p = 0.42). We found no evidence that the association between DM and TB relapse was modified by time to relapse (p for effect modification: 0.79) (Fig. 2).

**Table 2:** Univariable and multivariable odds ratios for the associations between potential risk factors and TB relapse.

		Unadjusted odds ratio (95% CI) (n = 600)	*P*	Adjusted odds ratio (95% CI) (n = 502) *	*P*
DM		1.67 (1.18–2.38)	*0.004*	1.96 (1.22–3.15)	*0.005*
Age (year)	<40	Reference		Reference	
	40–59	1.44 (0.92–2.26)	*0.11*	0.88 (0.48–1.60)	*0.67*
	≥60	1.48 (0.98–2.23)	*0.06*	1.07 (0.62–1.84)	*0.82*
Body weight (kilogram)†	<50	Reference		Reference	
	50–69	0.90 (0.63–1.28)	*0.56*	0.53 (0.33–0.85)	*0.01*
	≥70	1.37 (0.80–2.35)	*0.26*	0.73 (0.37–1.45)	*0.37*
Sex	male	1.41 (0.99–2.00)	*0.06*	1.41 (0.86–2.31)	*0.17*
Indigenous population		5.17 (2.16–12.38)	*<0.001*	4.24 (1.56–11.54)	*0.01*
History of alcohol use		2.39 (1.51–3.77)	*<0.001*	1.79 (0.97–3.33)	*0.06*
History of smoking		1.54 (1.10–2.15)	*0.01*	1.17 (0.71–1.93)	*0.54*
Cancer		1.52 (0.90–2.58)	*0.12*	1.77 (0.96–3.27)	*0.07*
ESRD		1.50 (0.42–5.32)	*0.53*	1.37 (0.35–5.34)	*0.65*
Coexisting of extra-pulmonary lesion		1.08 (0.70–1.65)	*0.74*	1.16 (0.69–1.96)	*0.57*
Initial cavitation‡		1.40 (0.93–2.10)	*0.10*	1.02 (0.62–1.68)	*0.94*
Suboptimal regimen		1.55 (1.03–2.33)	*0.03*	1.52 (0.93–2.50)	*0.10*
DOT ≥60%		1.48 (1.01–2.17)	*0.05*	1.10 (0.69–1.75)	*0.70*

*Only case-c°ntrol pairs with complete information on all covariates were included in the multivariable analysis

† Missing information in 24 case-control pairs

‡ Missing information in 25 case-control pairs.

**Table 3:** The association of DM with TB relapse by different definitions for DM (n  =  251 pairs of cases and controls).

DM definition*	No. of cases with DM (%)	No. of controls with DM (%)	Adjusted OR for DM and TB relapse (95% CI)
Previous TB treatment	89 (35.5)	54 (21.5)	1.96 (1.22–3.15)
Previous TB treatment + first year after treatment completion	102 (40.6)	56 (22.3)	2.40 (1.49–3.86)
One year before relapse	99 (39.4)	57 (22.7)	2.33 (1.44–3.76)
Two years before relapse	102 (40.6)	60 (23.9)	2.23 (1.39–3.58)
Three years before relapse	103 (41.0)	62 (24.7)	2.14 (1.34–3.43)

* DM status ascertained by either medical chart review °r use of DM medication in NHI database.

The proportion of DM patients who received at least one measurement of HbA1c was 31.0% and 32.7% among cases and controls during previous anti-TB treatment. Assuming that measurement of HbA1c was a proxy for better glucose monitoring and access to diabetes care, we found that DM patients without any measurement of HbA1c had higher risk of relapse (adjusted OR: 1.98, 95% CI: 1.13–3.45) than those who received at least one measurement of HbA1c (adjusted OR: 1.62, 95% CI: 0.80–3.29) and those without DM (reference group).

Our multivariable analysis revealed that indigenous people had significantly higher risk for relapse than non-indigenous people, with an adjusted OR of 4.24 (95% CI: 1.56–11.54). Patients with moderate body weight (50–69 kg) during anti-TB treatment had lower risk for relapse (adjusted OR 0.53, 95% CI: 0.33–0.85) compared to those with low body weight (<50 kg). Alcohol use (OR 2.39, 95% CI: 1.51–3.77) and suboptimal anti-TB regimen (OR 1.55, 95% CI: 1.03–2.33) were associated with TB relapse in the univariable analyses but not in the multivariable analysis.

## Discussion

In this nationwide nested case-control analysis, we found that presence of DM during anti-TB treatment was associated with subsequent relapse of TB; the OR was 1.96 (95% CI: 1.22–3.15) after adjusting for demographic and clinical confounders. Importantly, over two-thirds of the DM-TB patients did not receive blood glucose monitoring using HbA1c during anti-TB treatment. The positive association between DM and relapse of TB and the inadequate monitoring of DM control in TB patients highlight the need to strengthen co-management of DM and TB in our study population.

The estimated odds ratio for the association between DM and relapse of TB in our study was lower compared to previous observational studies. The relative risk in previous studies ranged from 1.76 to 8.15, and a recent systematic review reported a pooled estimate of 3.89 (95% CI: 2.43–6.23) [7], [23]. We note that the numbers of relapse cases in previous studies were all very small, ranging from five to 107 (compared to 300 in the current analysis). Most of these studies did not consider confounding factors such as age, clinical and TB treatment-related factors, and the observed large effects in these studies could be due to the spurious association caused by uncontrolled confounding. In some studies, the diagnosis of DM might occur after TB relapse; therefore the observed association between DM and relapse of TB could come from reverse causation due to TB-induced transient hyperglycaemia. Moreover, the definition of TB relapse was not specified in previous studies, making it difficult to compare across studies. In our study, the attenuated association between DM and only patients with bacteriologic evidence of TB relapse may indicate atypical clinical presentations of DM-TB patients for the relapse episode. Statistics on atypical TB presentation and bacillary load among DM patients varies [24], but it has been shown that hosts with advanced immunosuppression, such as patients with comorbid HIV infection, are more likely to present with smear-negative or extra-pulmonary lesions [25], [26]. During follow-up of DM patients who completed anti-TB treatment, if atypical TB presentations occurred, diagnostic testing including biopsy may help in early identification of relapse and initiation of proper management.

In our age-specific analysis, the adjusted odds ratio for DM and relapse of TB attenuated in those older than 60 years of age. Similar finding was noted by Jeon et al in a systematic review on DM and the risk of incident TB [6]. One explanation for the stronger association in the younger population is that DM might be more associated with risk of TB infection/re-infection than risk of reactivation, since TB disease in the elderly (compared to younger population) is more likely due to reactivation than infection/re-infection. On the other hand, previous studies using molecular typing of TB strains suggested that reactivation (rather than re-infection) was responsible for the majority of recurrence during the initial period after anti-TB treatment completion [27], [28]. Our subgroup analysis provided no evidence that the association between DM and TB recurrence differed substantially by time since completion of previous treatment. In previous studies, risk of re-infection increased with time in low TB burden countries, whereas endogenous reactivation was more likely to occur in the initial period after treatment completion [27], [28]. Further studies are needed to understand whether the increased risk of TB recurrence in DM patients is mainly mediated through reactivation or re-infection, or both.

Another line of evidence on DM and TB came from studies on glycaemic control and TB risk. Improved glycaemic control (HbA1c <7.0%) was associated with lower incidence of TB in one cohort study [29], but the impact of glycaemic control on relapse of TB has not been studied. Because of insufficient information on glycaemic control in the study participants, we were not able to investigate whether adequate glycaemic control during anti-TB treatment would reduce the hazard of DM on TB relapse. Nonetheless, using measurement of HbA1c as a proxy for optimal glucose monitoring, we found that DM-TB patients without any measurement of HbA1c had a higher risk of relapse than those who received at least one measurement of HbA1c and those without DM. In the analysis of different exposure windows for DM, we found that the presence of DM, either before the completion of previous TB treatment or after treatment completion, was significantly associated with TB relapse. The result suggested that good glycaemic control should ideally be maintained even after treatment completion.

Previous animal studies found that DM hosts had higher bacterial load at infection [30], [31] and were more likely to have dysfunction in Th1 cell immunity and delayed responses to TB infection [32], [33]. A past human study also reported impaired chemotaxis of neutrophil to TB pathogens among DM patients [34]. All these studies support DM as a contributing factor for relapse of TB either through re-infection by a new TB strain or through reactivation of prior infection.

The strengths of our study include the use of nationwide TB registry to investigate the association between DM and relapse of TB, with much larger sample size of relapse cases than previous observational studies. We adjusted for a number of socio-demographic and clinical factors to avoid confounding bias. Our definition of DM was based on the national health insurance data and medical chart review; therefore we were unlikely to miss cases with diagnosed DM. Lastly, the diagnosis of relapse of TB was based on bacteriological and pathological evidence in order to minimize the chance of outcome misclassification.

Our study also has limitations. First, DM patients may have increased utilization of health care and were therefore more likely to receive diagnostic examination for TB. This would have overestimated the association between DM and TB relapse since health care utilization was not measured and adjusted for in our analysis. We compared the use of sputum acid fast stain smear among DM and non-DM patients without relapse, and found similar use of sputum smear in the two groups (median (IQR): 0 (0–0.59) vs. 0 (0–0.43) per year, p-value from Wilcoxon rank-sum test: 0.66). In addition, we found that receiving any testing for HbA1c was associated with a lower risk of relapse in our population. Therefore we believe the observed association between DM and TB relapse cannot be fully explained by this detection bias. Second, as in many other places, DM might be under-diagnosed in our study population. Since we measured the presence of DM before the occurrence of TB relapse, the under-diagnosis was unlikely to be differential between cases and controls. This non-differential misclassification of DM would bias our effect estimate toward the null. Third, the information on several confounding factors (e.g., smoking and alcohol use) was based on medical chart review and therefore might not necessarily reflect the actual exposure status. Similarly, we did not adjust for drug susceptibility status in our analysis because this information was not available for 51 case-control pairs (20.3% of total). When we restricted the study population to those with drug susceptibility information and adjusted for it in the multivariable analysis, the association between DM and TB relapse remained unchanged (adjusted OR  = 1.94, 95% CI: 0.9–4.18). In our main analysis, we excluded 49 case-control pairs because of missing information in body weight and results of chest X-ray. However, this will introduce selection bias only if the reason for selection is simultaneously related to both exposure and outcome of interest [35]. Although the missingness of weight information was likely to be related to DM, we do not believe that it would be related to the risk of TB relapse. Therefore the possibility of selection bias is small. Lastly, because most patients' bacterial isolates were not available from both their incident and relapsed episodes, we could not differentiate reactivation and re-infection by DNA fingerprinting. If additional DM-TB association studies were to be conducted, prospective study design with collection of bacterial isolates and molecular typing will be needed to distinguish between reactivation and re-infection among relapse patients.

Only one third of the DM-TB patients in our study received HbA1c monitoring during the entire course of anti-TB treatment, and it reflected that the situation of clinical practice for DM-TB patients had not followed the recommendation of testing HbA1c at least two times a year [36]. This is in sharp contrast with the high rate (70.9–84.6%) of receiving at least one measurement of HbA1c per year among general DM patients in Taiwan [37]. The low rate of glycaemic monitor among DM-TB patients could be explained by the fact that DM care has not been incorporated into TB care in Taiwan's DOT program. While WHO called for more collaboration between TB and DM care in 2011 [38], it has been estimated that between 66,500 to 1,225,000 US dollars per year would be needed to screen DM in TB cases of Southeast Asia, with additional 5 to 92 million dollars per year for DM care in TB cases [39]. Cost-effectiveness analyses for DM-TB co-management are therefore badly needed for policy makers to understand potential impact of this new intervention. The results of our study can be used to inform this type of analysis regarding the impact of co-management on TB relapse.

In conclusion, the presence of DM was independently associated with increased risk of subsequent TB relapse in this national cohort of TB patients. We recommend that, upon completion of anti-TB treatment, follow-up strategies targeting DM-TB patients must be strengthened to detect TB relapse early. Although further studies are needed to confirm whether improved glycaemic control decreases the risk of relapse, we urge that rigorous glucose control in DM-TB patients should be considered seriously by all TB programs. Inadequate blood glucose monitoring in TB patients in this national study highlights the gap between recommendation and current practice, an issue requiring urgent attention.
